# Community-acquired pneumonia associated with immunosuppression due to follicular thyroid cancer: a case report

**DOI:** 10.1186/s13256-024-04576-2

**Published:** 2024-05-26

**Authors:** Lilia Jannet Saldarriaga Sandoval, Modesta Alcántara Rojas, Fabiola Pinedo Idrogo

**Affiliations:** 1 Universidad Nacional de Tumbes, Tumbes, Peru; 2 Regional Hospital of Lambayeque, Chiclayo, Peru

**Keywords:** Community-acquired pneumonia, Cancer immunosuppression, Follicular thyroid cancer

## Abstract

**Background:**

We present the case of a woman with cancer, which weakened the immune system and increased the risk of infection. Thus, infections are a frequent complication of cancer. The development of community-acquired pneumonia, an acute respiratory infectious disease that damages the lung parenchyma, caused by the invasion of pathogenic microorganisms, can lead to respiratory failure with multiorgan failure due to respiratory sepsis.

**Case presentation:**

Case report of a 38-year-old mixed-race woman with diabetes mellitus and irregular treatment, who was admitted with community-acquired pneumonia complicated by type I respiratory failure requiring mechanical ventilation. During her hospital stay, she developed ventilator-associated pneumonia, recurrent empyema, bronchopleural fistula, refractory septic shock and multiorgan dysfunction despite multiple interventions. The patient required prolonged mechanical ventilation, vasopressor support and antibiotic therapy. After 62 days, metastatic papillary thyroid carcinoma was diagnosed. She presented with hypoparathyroidism and permanent hypocalcemia. She died after multiple complications and a refractory critical condition.

**Conclusion:**

The case exemplifies the potential severity of community-acquired pneumonia in a patient with risk factors such as diabetes and immunosuppression. It highlights the complexity of treating multiple comorbidities and the importance of multidisciplinary management with close surveillance for timely interventions for complications.

## Introduction

Contemporary medical management often confronts complex and multifaceted cases that challenge the diagnostic and treatment capabilities of the healthcare professionals [1]. People with cancer may have an increased risk of infections due to changes in the immune system that control their body’s defense systems, and can affect these systems in different ways, making people more prone to infection because the system is complex and uses the body to resist infection by germs, such as bacteria or viruses. Such is the case of community-acquired pneumonia (CAP), whose acute respiratory infection damages the lung parenchyma through the invasion of pathogenic microorganisms (viruses, bacteria, fungi and parasites) acquired outside the hospital environment [2].

In the present analysis, we address the case of a woman who, after 2 months of hospitalization, exhibited a sequence of remarkable medical complications. Initially, the patient was admitted due to community-acquired pneumonia, which evolved into type I respiratory failure, making intervention with mechanical ventilation imperative to ensure adequate oxygenation and pulmonary ventilation [3]. During the course of her hospitalization, suggestive clinical findings were noted in the cervical region, leading to a biopsy that revealed an additional diagnosis of thyroid cancer. This clinical case follows CARE guidelines; the inherent complexity of treating multiple diseases in one patient is highlighted, emphasizing the importance of a multidisciplinary approach and constant vigilance to recognize and optimally manage secondary complications [4]. We present the case below with a narrative review of the literature, attempting to characterize the clinical, physiological, therapeutic and prognostic aspects of this pathology.

## Narrative

### Clinical case

History, current disease and physical examination, 38-year-old mixed-race woman, from Bagua, housewife, with a pathological history of diabetes mellitus with irregular treatment, family member reported that the patient presented with abdominal pain of mild to moderate intensity, nausea, vomiting, an unquantified thermal rise that persisted and intensified associated with generalized tonic–clonic movements, and sensory impairment, so she went to the health center, where she was tested with an uncertain result. She was referred to a private clinic, where she remained 1 day with unfavorable evolution and was referred to the hospital emergency room,

Physical examination; head and neck: normocephalic, symmetrical, no palpable adenopathy, neurological status with disorientation and soporous, symmetrical thorax, rhythmic heart sounds, tachycardic, vesicular murmur passes in both lung fields, bibasal cramps, respiratory distress, dyspnea with oxygen support, with sustained low saturation, with reservoir mask at 15 L, soft abdomen, depressive, no palpable masses, limited mobility initially, was hospitalized due to community-acquired pneumonia, which progressed to type I respiratory failure, requiring mechanical ventilation to maintain respiratory homeostasis, admitted to the intensive care unit for acute respiratory failure and respiratory failure on mechanical ventilation secondary to pneumonia associated with mechanical ventilation due to *Klebsiella pneumoniae*. Evolution with refractory septic shock of respiratory focus, requiring initiation of norepinephrine, then epinephrine due to persistent hypotension.

Exploratory thoracotomy was performed, identifying a massive left pneumothorax with empyema, left pulmonary decortication and wedge resection of the anterobasal segment and lingula, with a thoracic drainage. The patient persisted with bronchopleural fistula, for which she underwent multiple reoperative procedures without resolution.

Subsequently, he presented new ventilator-associated pneumonia due to the *Pseudomona aeruginosa* and *Acinetobacter baumannii* complex, with a torpid evolution. She required to be prolonged mechanical ventilation and a tracheostomy.

Computed Axial Tomography of the thorax showed multiple lesions compatible with pulmonary metastasis. A thyroid biopsy was performed, which reported metastatic papillary thyroid carcinoma. The patient developed hypoparathyroidism and permanent, severe hypocalcemia.

After multiple complications, prolonged ventilatory support and a refractory critical condition with acquired weakness in the intensive care unit, the patient died after 62 days and was discharged from the intensive care unit to the medical service. After 5 days of stay, the tracheostomy was decannulated, the patient ventilated spontaneously, with an indication for consultation with oncology, palliative treatment was started, and the patient requested voluntary withdrawal to go home. This case report seeks not only to describe the clinical course and interventions performed, but also to highlight the diagnostic and therapeutic challenges inherent in the management of multiple concurrent diseases in a single patient.

### Complementary tests and evolution

She was admitted to emergency observation for hemodynamic and ventilatory monitoring and waiting to be transferred to the intensive care unit, due to multiorgan dysfunction syndrome secondary to bacterial versus viral community-acquired pneumonia, in addition to alterations in basal glucose with figures of 280 mg/dL, it was decided to intubate and place her on mechanical ventilation in Volume Control mode, assisted by FIO2 control: 55%, PEEP: 7, under sedoanelgesia, with an unfavorable evolution due to pulmonary emphysema, pneumonia associated with mechanical ventilation by various germs, complementary tests and diagnostic tests such as brain tomography without contrast, chest tomography, neck tomography, fibrobronchoscopy, electrocardiogram, abdominal ultrasound, liver and biliary tract ultrasound, neck ultrasound. The diagnostic tests performed allowed to have the diagnostic impressions of hydroelectrolytic disorders, alterations in blood gas and acid–base balance, coagulation profile, liver profile, as well as parenchymal consolidation associated with cavitation and areas of abscessed necrosis in the right lung, diffuse pulmonary nodules in both lung fields, later, tumor in right hepatic lobe papillary thyroid carcinoma stage IV metastatic to lung, bone and liver, post-surgical hypothyroidism due to radical thyroidectomy, permanent severe hypocalcemia due to panhypothyroidism.

From the onset of the disease to the follow-up of the patient this is detailed in the following Table 1.

**Table 1:** Evolution and follow-up of the patient. Source: medical history evolution data

Event	Date and time	Description
Initial presentation	*T* = 0	Patient presents with symptoms specific to clinical picture of mild to moderate abdominal pain, nausea, vomiting, thermal rise
Start of treatment	*T* = 7 days	Treatment is prescribed to address symptoms with unfavorable evolution with respiratory distress progressing to type I respiratory failure, culminating in mechanical ventilation therapy complicated by *Klebsiella pneumoniae* infection
Follow up	*T* = 1 mes	The patient evolves with refractory septic shock of respiratory focus, requiring initiation of norepinephrine, then epinephrine due to persistent hypotension and sedoanelgesia. Fibrobronchoscopy was performed with bilateral atelectasis, sampling for crops
Follow up	*T* = 3 meses	Patient admitted to the operating room with a diagnosis of empyema and bronchopleural fistula, exploratory thoracotomy was performed, identifying massive left pneumothorax with empyema, left pulmonary decortication and wedge resection of anterobasal segment and lingula, with thoracic drainage Patient presented septic shock due to bronchial secretion culture of *Acinetobacter baumanni* Complex. Tumor is located on the left side, hard consistency, non-mobile, no impression of pain on palpation Thyroid carcinoma with lymph node metastasis is diagnosed with indication for I131 screening and treatment with I131
Further evaluation	*T* = 6 months	Patient undergoes thyroidectomy for thyroid neoplasia due to thyroid biopsy Surgical risk tests are performed and tracheostomy is placed, and fibrobronchoscopy is performed for follow-up to evaluate the long-term efficacy of treatment by first receiving treatment for thyroid cancer and with favorable evolution is discharged from ICU to medicine service The tracheostomy was removed after 118 days and the patient was able to ventilate spontaneously without complications
Final evaluation	*T* = 1 year	Patient begins palliative outpatient treatment of cancer 3 months after voluntary withdrawal from hospitalization to continue treatment at home for metastases in abdomen, liver and bones until death

*T* time

## Discussion

The present case illustrates the torpid evolution of a patient with in-hospital pneumonia complicated with empyema and recurrent bronchopleural fistula, refractory septic shock and multiorgan dysfunction. Diabetes mellitus is a disease that can compromise the immune system and increase the risk of infections, including pneumonia. If the patient has irregular treatment for diabetes, this could contribute to suboptimal immunosuppression and increased susceptibility to pneumonia.

Pneumonia associated with mechanical ventilation (VAP) is a frequent complication in critical patients, with a reported incidence between 9 and 27% [5]. In the patient’s case, VAP due to *Klebsiella pneumoniae* rapidly evolved into severe sepsis, a situation described in 20% of cases [6]. Immunosuppression associated with diabetes mellitus and other clinical factors may increase the risk of infections, including pneumonia.

Several studies have shown that immunosuppression in patients with pneumonia, whether due to medical conditions such as diabetes mellitus or immunosuppressive treatments, increases the risk of severe respiratory infections, including pneumonia [7, 8]. In the patient’s case, her immunosuppressed state due to irregularly treated diabetes mellitus could have contributed to her susceptibility to severe respiratory infection. One of the most feared complications of VAP is empyema, reported in 2–12% of cases, which the patient presented with [9]. In this case she required multiple surgical interventions without achieving resolution of the bronchopleural fistula, probably due to extensive necrosis and infection of the pulmonary parenchyma.

Prolonged mechanical ventilation is associated with progressive deterioration of pulmonary function. In this case, tracheostomy and ventilatory support was required for 62 days, which correlates with higher mortality [10]. On the other hand, septic shock has a high morbimortality, estimated at 20–50% [11]. He developed multiple organ dysfunction despite antibiotic therapy and support with two vasopressors, which denotes the refractoriness of the condition. The literature supports the importance of timely evaluation and management to improve clinical outcomes in these patients.

In addition, timely diagnosis and treatment are crucial to improve clinical outcomes in these patients. In the patient’s case, her immunosuppressed status due to diabetes mellitus and other clinical factors could increase her risk of pneumonia-related complications. Proper evaluation and management of her immunosuppressed condition are essential to optimize her prognosis. Optimal glycemic control and proper management of diabetes mellitus are critical to prevent acute and chronic complications, including infections and deterioration of overall health status. The patient has a history of diabetes mellitus with irregular treatment, which could compromise her immune status and increase her susceptibility to infections such as pneumonia. A comprehensive approach to diabetes management, including glycemic control and regular follow-up, is crucial to improve your health status and prevent future complications. Current guidelines emphasize the importance of optimal glycemic control and comprehensive management of diabetes mellitus to prevent acute and chronic complications, including infections [12, 13]. The patient’s varied clinical presentation requires a broad differential diagnostic approach that considers multiple medical conditions, including infectious and non-infectious diseases [14]. Finally, the presence of comorbidities such as thyroid carcinoma and hypoparathyroidism contributed to the fatal outcome, exemplifying the potential severity of VAP in a critically ill patient.

## Conclusion

This report describes the case of a 38-year-old female patient with a history of poorly controlled diabetes mellitus who developed community-acquired pneumonia complicated by acute respiratory failure and admission to intensive care. The torpid course, characterized by ventilator-associated pneumonia, recurrent empyema, bronchopleural fistula, refractory septic shock and multiorgan dysfunction despite all measures instituted, highlights the potential severity of this condition.

The coexistence of this malignant disease with immunosuppression may compromise the patient’s immune response, predisposing him or her to severe respiratory complications such as pneumonia. Although diabetes mellitus was not a factor directly implicated in this case, it is crucial to note that its presence can further exacerbate the risk of complications and the severity of pneumonia.

The case also evidences the challenges inherent in the diagnosis and management of multiple concurrent comorbidities in a critically ill patient. The late diagnosis of metastatic thyroid carcinoma and the appearance of hypoparathyroidism and hypocalcemia accentuated the complexity of the picture.

The importance of maintaining a high index of clinical suspicion and a multidisciplinary approach that allows the recognition and timely treatment of associated complications is emphasized. Strict glycemic control and comprehensive management of comorbidities such as diabetes mellitus are essential to optimize outcomes. Therefore, in patients with follicular thyroid cancer and other immunosuppression, close surveillance should be maintained for early detection and appropriate management of respiratory complications, including pneumonia, with a multidisciplinary approach that addresses both the underlying disease and associated comorbidities. This case highlights the need for early prevention, diagnosis and treatment protocols for community-acquired pneumonia and its possible complications, especially in patients with risk factors such as underlying immunosuppressive diseases.

## Data Availability

Not applicable.
